# Mechanisms of Carotenoid Intestinal Absorption: Where Do We Stand?

**DOI:** 10.3390/nu11040838

**Published:** 2019-04-13

**Authors:** Emmanuelle Reboul

**Affiliations:** Aix-Marseille University, INRA, INSERM, C2VN, 13005 Marseille, France; Emmanuelle.Reboul@univ-amu.fr; Tel.: +33-4-91-324-278

**Keywords:** carotenes, xanthophylls, bioavailability, intestine, membrane transporters

## Abstract

A growing literature is dedicated to the understanding of carotenoid beneficial health effects. However, the absorption process of this broad family of molecules is still poorly understood. These highly lipophilic plant metabolites are usually weakly absorbed. It was long believed that β-carotene absorption (the principal provitamin A carotenoid in the human diet), and thus all other carotenoid absorption, was driven by passive diffusion through the brush border of the enterocytes. The identification of transporters able to facilitate carotenoid uptake by the enterocytes has challenged established statements. After a brief overview of carotenoid metabolism in the human upper gastrointestinal tract, a focus will be put on the identified proteins participating in the transport and the metabolism of carotenoids in intestinal cells and the regulation of these processes. Further progress in the understanding of the molecular mechanisms regulating carotenoid intestinal absorption is still required to optimize their bioavailability and, thus, their health effects.

## 1. Introduction

Carotenoids are hydrophobic molecules synthesized by plants and by some microorganisms (bacteria, algae, or fungi). Carotenoid physicochemical properties determine their distribution in the cellular environment: carotenoids are associated with membrane lipid bilayers and cytosolic lipid droplets. More than 600 different carotenoids have been found in nature, but only 40 in the human diet and about 20 have been clearly identified in human blood and tissues [1,2].

Carotenoids are polymers of isopentenyl diphosphate with 40 carbon atoms. They derive chemically from a basic structure formed by the linear sequence of 8 isoprenic units, associated in two groups of four units head to tail. The first molecule of this biosynthesis pathway is phytoene, which presents 3 conjugated double bonds in the center of the molecule and 6 other unconjugated double bonds through the length of the molecule. Phytoene is then enzymatically desaturated to produce phytofluene and eventually lycopene, a linear basic structure (C40H56) with many conjugated double bonds showing a characteristic red color [3]. Other carotenes derive from lycopene by cyclization and dehydrogenation, and xanthophylls derive from carotenes by oxidation [4] (Table 1). Some xanthophyll carotenoids such as β-cryptoxanthin [5] or lutein [6] can be found in esterified forms. Additionally, each carotenoid double bond can take a *trans* or *cis* configuration. Most of natural carotenoids are *all-trans* molecules, but *cis*-isomers can be produced during heat treatments [7]. Finally, it is worth mentioning that small amounts of apocarotenoids, i.e., cleavage products of parent 40-C carotenoids, can be naturally found in foods and/or produced during food processing, but usually represent less than 5% of the parent carotenoid levels [8,9].

The main dietary sources of carotenoids are colored fruits and vegetables (Table 1).

Carotenoid health properties were initially mainly accredited to their antioxidant properties as carotenoid are, at least in vitro, powerful radical quenchers [12]. Later, new investigations have highlighted the carotenoid ability to regulate intracellular signalling cascades, thus influencing both gene expression and protein translation in a broad number of metabolic pathways related to inflammatory and oxidative stress modulation [13]. However, the physiological relevance of these observations still needs to be fully established in humans. Interestingly, randomized placebo-controlled clinical trials have evidenced that supplementation with the xanthophylls lutein and zeaxanthin, which specifically accumulate into the human macula, was associated with improved visual function and decreased risk of progression to late age macular degeneration. These xanthophylls also display encouraging preventive and therapeutic effects on cataracts and retinopathies [14]. Finally, some carotenoids, such as α-carotene, β-carotene and β-cryptoxanthin, are vitamin A precursors. Indeed, they can be cleaved, mainly at the intestinal level, and metabolized into retinol (cf. Section 3.2).

After a short description of carotenoid fate in the human upper gastrointestinal lumen, a focus will be put on the identified proteins participating in carotenoid transport and metabolism in intestinal cells and on the regulation of these processes.

## 2. Digestion Process of Carotenoids

Fat-soluble micronutrients, including carotenoids, follow the fate of lipids in the human upper digestive tract. The first step of their digestion is thus their dissolution in the fat phase of the meal [15,16]. This phase is emulsified into lipid droplets in the stomach and duodenum.

The hypothesis that a carotenoid *cis*-isomerization could take place during gastric digestion was emitted [17], but finally refuted by a study in humans [16]. Recently, in vitro digestion experiments mimicking duodenal conditions showed that no significant isomerization of lycopene, β-carotene, or lutein occurred either [18].

It has been suggested that xanthophyll ester hydrolysis by lipases is indispensable prior to absorption. The cholesterol ester hydrolase (CEH) from pancreatic juice is likely responsible for the release of free xanthophyll from xanthophyll esters [5]. The remaining xanthophyll esters, if any, may either be cleaved at the brush border level or enter the enterocyte to be hydrolyzed in the cytosol [19].

During duodenal digestion, carotenoids are incorporated with other lipids (i.e., cholesterol, phospholipids) and lipid digestion products (i.e., free fatty acids, monoacylglycerols, lysophospholipids) into mixed micelles [20]. A fraction of carotenoids may also associate with proteins. For instance, the milk lipocalin β-lactoglobulin is able to bind β-carotene and does not alter its absorption compared to mixed micelles [21]. However, the mechanisms of carotenoid absorption may depend on carotenoid binding vehicles. Mixed micelles are likely isolated from the rest of the bolus in the unstirred water layer of the glycocalyx area and approach the brush border membrane [22] where carotenoids can be absorbed by passive diffusion and/or via a transporter-dependent process (see Section 3).

Carotenoid bioaccessibility (i.e., the fraction of carotenoids released form their food matrix and included in mixed micelles—which represents the fraction of carotenoids potentially able to be absorbed by the intestine) is highly variable. An in vitro digestion study highlighted that lycopene bioaccessibility was very limited (from 0.1% in raw tomatoes to 1.5% in tomato puree), β-carotene bioaccessibility was fairly low (from about 4% in carrot puree to 14% in carrot juice), while lutein bioaccessibility was the highest (from 37% in raw spinach leaves to 48% in boiled spinach). These values correlate with in vivo data and highlight the fact that the disruption of the food matrix by thermal treatment or processing can increase carotenoid bioaccessibility [10]. Xanthophylls were consistently shown to display a higher bioaccessibility than carotenes in different studies [10,11], probably because the presence of one or two hydroxylated group(s) increases their solubility into the micellar structures. Interestingly, phytoene and phytofluene also displayed a very high bioaccessibility. This may be linked to their more flexible molecular structure, compared to other carotenoids, which likely increase their incorporation into mixed micelles as well [11].

## 3. Carotenoid Absorption through the Enterocyte

Absorption efficiency of labelled β-carotene is widely variable among clinical studies, fluctuating from ≈3% to 80%, but usually ranging from 10% to 30% [23,24]. This can partly be due to the variable bioaccessibility of β-carotene (see above), but it may also reflect its moderate uptake and transport through the enterocyte. It should be mentioned that β-carotene absorption efficiency was usually measured following a single meal. However, the intestine can store β-carotene from a first meal to release it during subsequent postprandial phases in humans [25]. β-carotene absorption efficiency may, thus, be underestimated in some trials.

Studies using differentiated Caco-2 cell monolayers showed that phytofluene, β-carotene, and lutein uptakes were similar and significantly higher than that of phytoene, while lycopene uptake was the lowest [26,27]. In the same way as bioaccessibility, uptake efficiency thus seems to correlate with carotenoid polarity and flexibility. This may be explained by the fact that polar and flexible carotenoids present a better affinity for lipid transporters and/or for plasma membranes, which would lead to an increased absorption.

### 3.1. Apical Transport Across the Brush Border Membrane of the Enterocyte

Carotenoid uptake by the enterocytes has been considered to occur by passive diffusion for four decades, which was inconsistent with the high inter-individual variability in absorption observed in humans, as well as with the isomer selectivity and the competition for absorption between carotenoids and other fat-soluble micronutrients observed at the intestinal level (see [20] for review). Different teams started to re-explore carotenoid absorption mechanisms in the 2000s and several lipid transporters playing a role in carotenoid uptake by the intestinal cell have since been identified.

A first critical result was the identification of the gene ninaD encoding a class B scavenger receptor, essential for xanthophyll cellular distribution in *Drosophila* [28]. In 2005, we then identified the Scavenger Receptor class B type I: SR-BI as a key transporter of lutein in human intestinal Caco2 TC7 cells. This ubiquitous transmembrane glycoprotein found at the apical membrane of the enterocytes is expressed following a decreasing gradient from the duodenum to the colon [29]. Intestinal SR-BI was shown to facilitate the uptake of free cholesterol, but also of other lipids such as cholesterol esters, phospholipids, and triacylglycerol hydrolysis products, thus presenting a low substrate specificity [30,31]. The effective role of SR-BI in terms of cholesterol transport is still subject to debate [32] and SR-BI was recently presented as a cholesterol sensor [33], regulating chylomicron secretion [34]. Its involvement in the intestinal uptake of carotenoids has been extended to lycopene [35], provitamin A carotenoids [36], as well as to phytoene and phytofluene [27]. As SR-BI is also involved in the uptake of vitamin D [37], E [38], and K [39], in cultured cells and in mice, we suggest that another primary role of SR-BI in the gut is the transport of minor molecules, such as fat-soluble vitamins and carotenoids. However, we specifically showed, using both Caco2 cells and transfected HEK cells, that SR-BI was not involved in the uptake of micellar preformed vitamin A (retinol) [36].

Another pervasive scavenger receptor of interest is CD36 (CD 36 molecule). This membrane protein is highly expressed at the brush border level of the duodenum and the jejunum [40]. It is supposed to play a key role in the intestinal uptake of long-chain fatty acids [41], but also displays a broad substrate specificity [42,43]. Recently, CD36 has been described as a lipid sensor and its impact on chylomicron secretion has been established in many studies [44]. Besides, CD36 facilitates, directly or indirectly, fat-soluble vitamin uptake in the intestine [37,39,45]. CD36 was also shown to facilitate the uptake of lycopene, β-carotene, α-carotene, β-crypthoxanthine, and lutein, but not that of phytoene and phytofluene, in transfected Griptite cells and/or cultured adipocytes [27,36,46]. This result was confirmed ex vivo for β-carotene using brush-border membrane vesicles from CD36-deficient and wild-type mouse intestines [47].

A last candidate for carotenoid uptake is the NPC1-like transporter 1 (NPC1L1), which is a major sterol transporter in the intestine [37,48]. NPC1L1 was suggested to be involved in α-carotene, β-carotene, β-cryptoxanthin, and lutein intestinal uptake [49,50], but not in that of lycopene, phytoene, and phytofluene [27,35].

It is still possible that a fraction of carotenoid is absorption via a passive diffusion process, depending on the carotenoid concentration in the lumen. We previously showed in Caco-2 cells that vitamin D absorption is carrier-mediated at physiological concentrations and occurs by passive diffusion at pharmacological concentrations [37]. We suggest that a similar phenomenon occurs for carotenoids.

Recently, we showed that a fraction of phytoene and phytofluene taken up by the intestinal cells could be effluxed back to the lumen [27]. This phenomenon was previously acknowledged for fat-soluble vitamins such as vitamin D, E, and K and was shown to be, at least partly, SR-B-dependent [37,38,39]. This efflux may contribute to the limited absorption efficiency of carotenoids. Further research is needed to clearly identify the membrane transporters participating in this pathway. Besides SR-BI, ABCB1 (ATP binding cassette B1, also known as P-glycoprotein) and ABCG transporters, such as ABCG5, appear as good candidates. Indeed, a recent study combining in silico, cell culture, animal, and genetic approaches showed that ABCB1 was involved in vitamin D intestinal efflux [51]. Additionally, polymorphisms in the *ABCG5* gene tended to contribute to individual response to lutein supplementation in humans [52].

### 3.2. Cytosolic Transport and Intracellular Metabolism

No carotenoid carrier protein has clearly been identified in the human gut so far. However, the lutein-binding protein HR-LBP (Human Retinal Lutein-Binding Protein) present in the human retina cross-reacts with antibodies raised against a carotenoid-binding protein present in the *Bombyx mori* midgut [53], suggesting that it could be an intestinal intracellular transporter of xanthophylls [54]. As carotenoid membrane transporters SR-BI, CD36, and NPC1L1 can traffic in the enterocyte, especially after a fat load, we previously suggested that they may act as cytosolic carotenoid transporters [20]. However, this hypothesis still remains to be verified. Similarly, the association found between a genetic variant in the Intestinal Fatty-Acid Binding Protein (IFABP) and the fasting plasma lycopene concentrations in humans [55] still need to be challenged to assess whether IFABP is actually a carotenoid-carrier.

Up to 40% of absorbed carotenoids remain unmetabolized [56]. β-carotene can be cleaved into retinal by a cytosolic enzyme, BCO1 (β-carotene oxygenase 1), via a one-step process in the enterocyte [57]. β-Carotene “low-converter” phenotypes, which have been reported in several clinical studies [58], are likely due to genetic variation in *BCO1* gene. Other provitamin A carotenoids, such as β-crypthoxanthin, can be cleaved into retinal though a multi-step process involving both mitochondrial BCO2 (β-carotene oxygenase 2) and cytosolic BCO1 [59]. The produced retinal is subsequently converted into retinol and esterified into retinyl esters by the lecithin:retinol acyltransferase (LRAT) and probably by the diacylglycerol acyltransferase 1 (DGAT1) that displays an acyl-CoA:retinol acyltransferase activity [60,61]. Both provitamin A and nonprovitamin A carotenoids can also be cleaved asymmetrically in apocarotenoids by BCO2 [62]. However, a recent study showed that only traces of asymmetric [^13^C]-β-apo-carotenals were found in plasma after [^13^C]-β-carotene ingestion, suggesting a lack of significant postprandial intestinal BCO2 activity in healthy humans [63].

No *cis-trans* isomerization of β-carotene was measured in intestinal cultured cells [26]. As *cis-*isomerization does not occur in the gastrointestinal lumen (see above), the site of the 9-*cis* isomerization of β-carotene reported in vivo [64] remain undetermined. Conversely, lycopene isomerization in *cis*-isomers was identified to occur at the enterocyte level [65].

**Figure 1:**
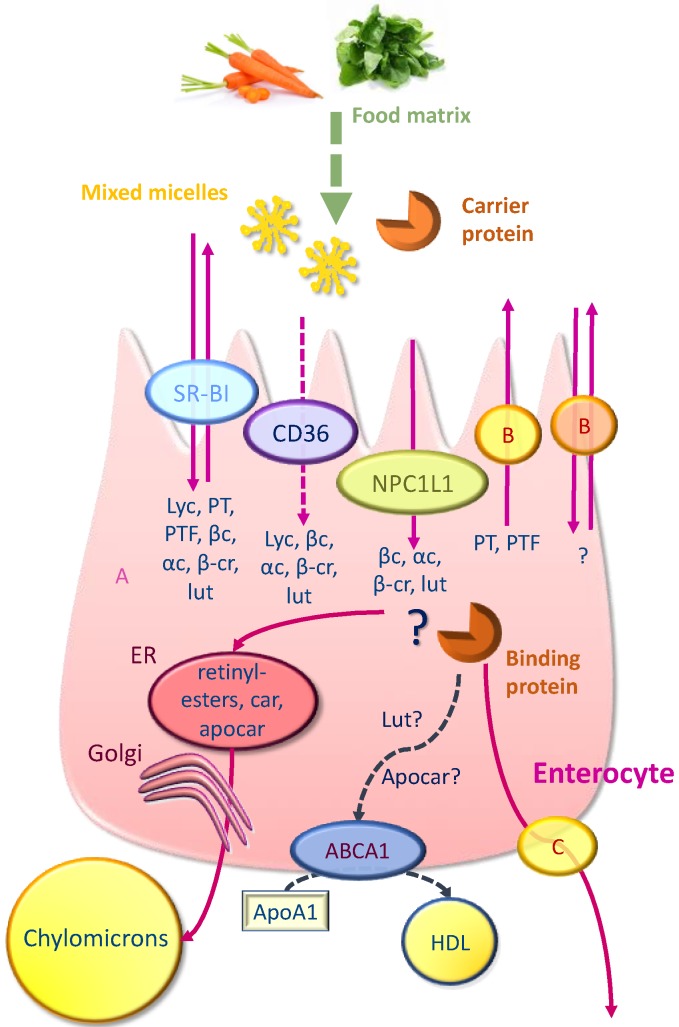
Uptake, transport, and secretion pathways of carotenoids across the enterocyte. PT = phytoene; PTF = phytofluene; Lyc = lycopene; βC = β-carotene; αC = α-carotene; βCr = β-cryptoxanthine; Lut = lutein; Car = carotenoids; Apocar = apocarotenoids; A = passive diffusion; B = unidentified apical transporter; C = unidentified basolateral efflux transporter; ? = putative pathway, and ER = endoplasmic reticulum. Carotenoids are captured from mixed micelles and possibly from carrier proteins by apical membrane transporters SR-BI, CD36, and NPC1L1. A fraction of PT and PTF can then be effluxed back to the intestinal lumen via apical membrane transporters (likely SR-BI and possibly other transporters). Another fraction is transported to the site where they are incorporated into chylomicrons. Some proteins may be involved in intracellular transport of carotenoids, but none has been clearly identified. Provitamin A carotenoids are partly metabolized into retinyl-esters. Retinyl-esters and carotenoids are secreted in the lymph into chylomicrons, while a part of xanthophylls and a part of the more polar metabolites, such as some apocarotenoids, may be secreted via an HDL pathway.

A summary of carotenoid transport pathways across the enterocyte is depicted in Figure 1.

### 3.3. Secretion Through the Basolateral Membrane of the Enterocyte

During the postprandial period, the major fraction of free carotenoids and retinyl esters originating from provitamin A carotenoid cleavage are packaged into chylomicrons (apoB-dependent pathway) that are secreted into the lymph to further join the bloodstream [20]. A non-apoB-dependent pathway (via high-density lipoproteins, HDL), mediated by the ABCA1 transporter, has been involved in vitamin E absorption [66] and possibly allows a part of free retinol absorption [20]. This HDL pathway may also exist for xanthophylls, such as lutein and zeaxanthin [67], but has not been proven to occur for other carotenoids. However, recent studies showed that several genetic variants in *ABCA1* gene were associated with lycopene [68], β-carotene [69], and lutein [70] postprandial responses in healthy subjects. Thus, further research is needed to fully understand the contribution of the intestinal HDL pathway to carotenoid absorption in humans.

## 4. Regulation of Carotenoid Transporter Expressions in the Enterocyte

Crucial factors modulating the expression and/or the activity of intestinal proteins involved in carotenoid absorption are provitamin A carotenoids, through a feedback regulation. Indeed, studies have pointed out that SR-BI activity is partly controlled by retinoids. Using both mouse models and human cell lines, it was specifically shown that retinoic acid produced from dietary precursors by BCO1 induced the expression of the intestinal transcription factor ISX that repressed the expression of both BCO1 [71] and SR-B1 [72], thus impacting both carotenoid conversion and uptake [73].

Additionally, many dietary factors other than retinoids were shown to regulate transporter expression in the intestine and may, thus, indirectly impact on carotenoid absorption.

Among these dietary factors, fat and fatty acids seem to play major roles. For instance, SR-BI expression in Caco-2 cells is increased by micellar oleic and ecosapentaenoic acids [74]. Conversely, CD36, NPC1L1, or ABCA1 expressions in rodent intestines are downregulated by dietary fat, including oleic acid [75,76] and cholesterol [77]. Such downregulation in NPC1L1 and ABCA1 expressions was also found after exposure of cultured intestinal FHs 74 or Caco-2 cells to phytosterols [78,79] and to long-chain polyunsaturated fatty acids [74,80].

In addition, dietary glucose increases SR-BI expression in both Caco-2 cells and mouse intestines [81] and decreases Caco2 cell ABCA1 expression [82].

Finally, some polyphenols were shown to decrease both SR-BI, NPCL1, and ABCA1 expressions in Caco-2 cells [83,84] and so did the cholesterol-lowering drug ezetimibe [49].

Host factors can also regulate carotenoid transporters. Among them, insulin resistance increases SR-BI intestinal expression in hamsters [85]. SR-BI post-transcriptional regulation also seems be dependent on bile secretion, with bile salts leading to a rise of intestinal SR-BI expression in rodents [86]. Finally, NPC1L1 expression was increased by estrogen [87] and cholecystokinin [88] in mouse intestines, while its expression was decreased by peptide YY in Caco-2 cells [89] (see [90] for review).

As the above results were exclusively obtained in cultured cells and animal studies, further investigations are deeply needed to address their relevance in humans.

## 5. Conclusions

To conclude, the understanding of carotenoid intestinal absorption by the intestine is far from being fully understood. Proteins including lipid membrane transporters (i.e., SR-BI, CD36, NPC1L1), the cleavage enzyme BCO1, and the transcription factor ISX have been showed to play important roles in carotenoid intestinal uptake and metabolism, but other proteins likely remain to be identified.

Genome-wide association studies (GWAS) and candidate gene association studies have identified correlations between single nucleotide polymorphisms in *SCARB1* (encoding SR-BI), *CD36*, *NPC1L1*, *BCO1*, and *ISX* and carotenoid blood concentrations. Interestingly, these studies also highlighted the impact of polymorphisms in genes encoding proteins likely indirectly linked to carotenoid metabolism (i.e., ELOVL fatty acid elongase 2). The involvement of such proteins in carotenoid intestinal metabolism still needs to be defined [91].

Carotenoid “low responder” or “high responder” phenotypes presumably correspond to individuals bearing associations of several “disadvantageous” or “advantageous” polymorphisms, respectively. In the future, it would thus be of major interest to take into account the carotenoid “low responder” or “high responder” phenotypes that are due to different transport and/or conversion efficiency, to propose tailored dietary recommendations to individuals and to thus optimize carotenoid health benefits.

## Figures and Tables

**Table 1:** Main dietary carotenoids.

Carotenoids	Molecular Structure	Examples of Food Sources (mg/100 g) [10,11]
Phytoene	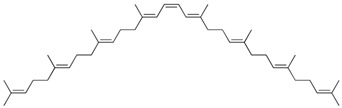	Tomato juice: 2.24 Carrot juice: 0.94
Phytofluene	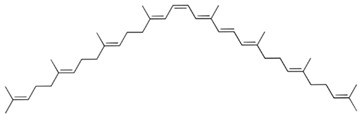	Tomato juice: 0.86 Carrot juice: 0.59
Lycopene	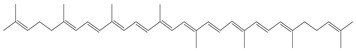	Tomato sauce: 15.92 Tomatoes: 3.03 Watermelon: 4.87
β-carotene	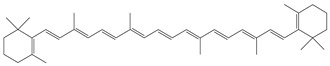	Raw carrot: 8.84 Canned carrot: 5.78 Cooked spinach: 5.24
α-carotene	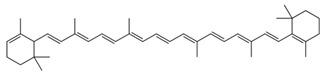	Carrot juice: 1.70
β-cryptoxanthin	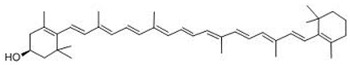	Sanguinello juice: 0.02
Lutein	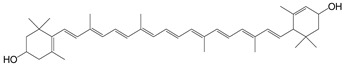	Cooked spinach: 7.04 Lettuce: 2.64

## Abbreviations

ABCA1	ATP binding cassette A1
ABCB1	ATB binding cassette B1
ABCG5	ATP binding cassette G5
BCO1	β-carotene-oxygenase 1
BCO2	β-carotene-oxygenase 2
CD36	CD36 molecule
CEH	cholesterol ester hydrolase
DGAT1	diacylglycerol acyltransferase 1
HR-LBP	human retinal lutein-binding protein
ISX	intestine-specific homebox
FABP	fatty-acid-binding protein
HDL	high-density lipoproteins
LRAT	lecithin retinol acyltransferase
NPC1L1	NPC1-like transporter 1
SR-BI	scavenger receptor class B type 1
